# Chair- and V-Shaped of H-bonded Supramolecular Complexes of Azophenyl Nicotinate Derivatives; Mesomorphic and DFT Molecular Geometry Aspects

**DOI:** 10.3390/molecules25071510

**Published:** 2020-03-26

**Authors:** Omaima A. Alhaddad, Khulood A. Abu Al-Ola, Mohamed Hagar, Hoda A. Ahmed

**Affiliations:** 1 Chemistry Department, College of Sciences, Al-Madina Al-Munawarah, Taibah University, Al-Madina 30002, Saudi Arabia; OHADDAD@taibahu.edu.sa (O.A.A.); Kabualola@taibahu.edu.sa (K.A.A.A.-O.); 2 Faculty of Science, Chemistry Department, Alexandria University, Alexandria 21321, Egypt; 3 Faculty of Science, Department of Chemistry, Cairo University, Cairo 12613, Egypt

**Keywords:** chair and V-shaped liquid crystals, supramolecular H-bonding, Azo mesogen, molecular geometry, nematic stability, DFT calculations

## Abstract

New geometrical architectures of chair- and V-shaped supramolecular liquid crystalline complexes were molded through 1:1 intermolecular hydrogen bonding interactions between 4-(4-(hexyloxy)phenylazo)methyl)phenyl nicotinate and 4-alkoxybenzoic acids. The length of terminal alkoxy acid chains varied, *n* = 6 to 16 carbons. The mesomorphic behaviour of these complexes was examined through differential scanning calorimetry (DSC) and polarizing optical microscopy (POM). Fourier-transform infrared spectroscopy (FT-IR) was carried out to confirm the presence of Fermi bands that appeared for the hydrogen bonding formation. Enantiotropic nematic phases were observed and covered all lengths of alkoxy chains. The geometrical structures of the prepared supramolecular complexes geometries were estimated by Density functional theory (DFT) calculations. The supramolecular complexes **I/An** are projected to exhibit a nonlinear geometry with V-shaped and chair-shaped geometry. The chair-shaped conformers of **I/An** were found to be more stable than V-shaped isomeric complexes. Moreover, the effect of the change of the mesogenic core on the mesophase thermal stability (**T**_C_) has been investigated by a comparative study of the present azo supramolecular H-bonding LCs (SMHBCs) **I/An** and our previously reported their Schiff base analogue complexes, **II/An**. The findings of the DFT illustrated the high impact of CH=N as a mesogenic core on the mesomorphic behavior in terms of the competitive lateral and terminal intermolecular interactions as well as the molecular electrostatic potential (MEP).

## 1. Introduction

Today, liquid crystalline compounds have been recognized as a one class of functional materials that combine mobility and molecular order with great potential to be used in sensors and bio-applications [1,2,3,4]. Moreover, the structural effect relationships could be a helpful tool to design a material to achieve desired properties for instrumental applications [5,6,7,8]. The new designing of functional materials to have novel molecular architectures is an important area of interest in material science and geometrical approaches [9,10]. Recently, azopyridine has been used in the formation of nano-fiber supramolecular self-assembling and hydrogen/halogen-bonding LCs with photo-induced transition phenomena [11,12,13,14,15,16,17]. They are particularly interesting due to their ability for trans-cis photo-isomerization molecular geometries upon irradiation with UV light [18]. In addition, the incorporation of the azobenzene moiety into the geometrical structure of liquid crystalline materials resulted in photo-switchable applications [2,19]. Further, azo derivatives have a wide variety of biological activities, optical switches, and nonlinear optics [1,20,21,22,23,24,25]. The combination of rigid and flexible segments (alkyl chains) in the molecular structures led to anisotropic architectures. Both permanent dipoles and polarizable moieties are essential for mesomorphism. Those changes in the characteristics of the LCs may impact the mesomorphic behaviour as well as the essential properties for technical uses. Recently, molecular interactions due to hydrogen bonding (H-bonding) formation have received more attention in the field of liquid crystal [26,27,28,29,30]. Supramolecular H-bonding LCs (SMHBLCs) designed through noncovalent interactions of suitable H-bond donors and acceptors are concerns of our area of interest [31,32,33,34,35,36,37]. Moreover, the mesomorphic characteristics of SMHBLCs can be finely tuned by modifying the molecular structure of the starting molecules. Mutual influence of mesomorphic behaviors requires stimulated data about the energies of frontier molecular orbitals as well as the molecular geometries of the prepared materials. Recently, density functional theory (DFT) becomes an essential routine method to correlate the outcome theoretical parameters with the experimental findings [38,39,40,41].

Several H-bonded mesogenic complexes containing azo, ester, and Schiff base central linkages have been widely reported [42,43,44,45,46,47]. Most of them are based on the linear intermolecular H-bonding interactions [48,49,50,51,52,53], and there are an attractive area of interest toward the formation of nonlinear SMHBLCs [54,55,56]. Further, the terminal alkoxy chains have important roles in the temperature range and stability of the mesophase of the LC compounds. As the length of the terminal wings increases, the molecules tend to orientate in parallel arrangements and enhance the formation of the mesophase [57].

**Scheme 1:**
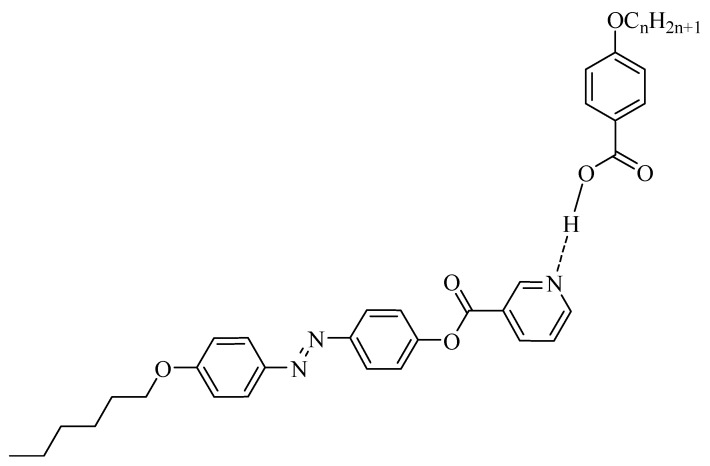
The prepared complexes **I/An**.

Herein, the aim of our work is to prepare a new homologues series of angular azo/ester linkage derivatives **I/An** through H-bonding interactions between 4-(4-(hexyloxy)phenylazo)methyl)phenyl nicotinate (**I**) and 4-*n*-alkoxybenzoic acids (**An**), and to analyze their thermal and mesomorphic behaviors with different lengths of the terminal alkoxy chains of the acid component, Scheme 1. We also aim to use the geometrical calculations to predict the structural and thermal parameters of individual components and their supramolecular complexes. Finally, a comparison is made between the present complexes and our previously reported Schiff base analogues to demonstrate briefly experimentally and theoretically in terms of the effect of the mesogen on the geometry as well as thermal parameters.

## 2. Experimental

### 2.1. Preparation of 4-(4-(Hexyloxy)phenylazo)methyl)phenyl Nicotinate

**Scheme 2:**
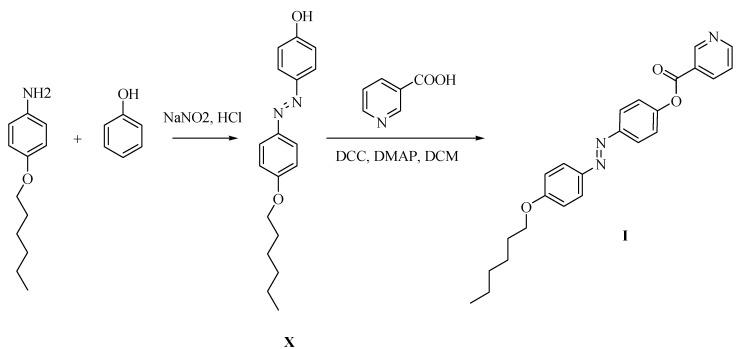
Preparation of4-(4-(hexyloxy)phenylazo)methyl)phenyl nicotinate (**I**).

The nitrogen base **I** has been prepared according to the following Scheme 2 [18,56].

### 2.2. Preparation of 1:1 Supramolecular H-bonded Complexes

**Scheme 3:**
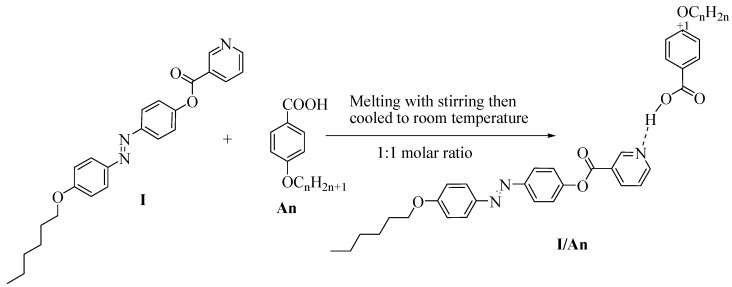
Preparation of 1:1 supramolecular H-bonding LCs (SMHB) complexes (**I/An).**

Equal molar ratios of both complementary components of supramolecular complexes (**I/An**) were melted until the intimate blend and then cooled to room temperature to give complexes in a crystalline powder state (Scheme 3).

## 3. Results and Discussion

### 3.1. FT-IR Conformation

**Figure 1:**
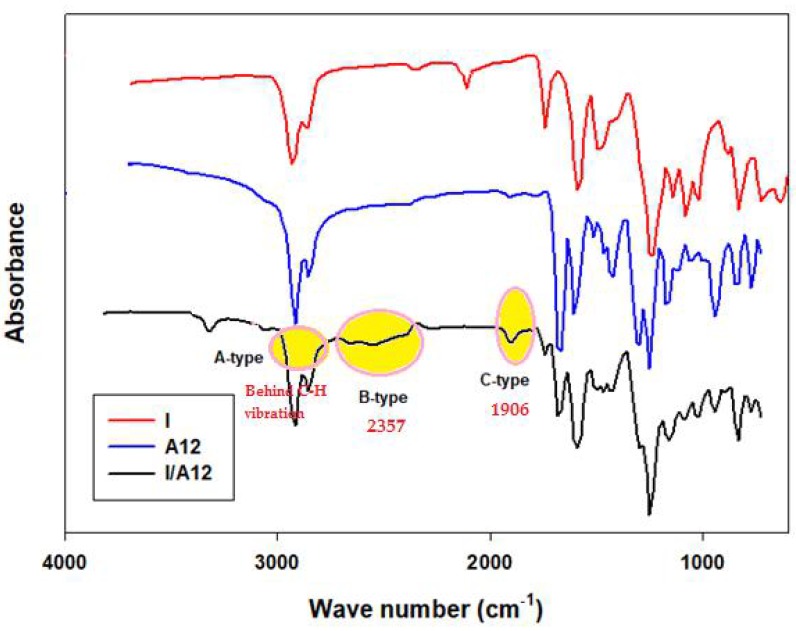
Fourier-transform infrared spectroscopy (FT-IR) spectra of **I, A12** and their SMHB complex **I/A12.**

It has been reported that [58,59,60,61] the *H*-bond formation can be approved by many tools such as X-ray, NMR, and IR spectroscopy. Fourier-transform infrared spectroscopy (FT-IR) spectral data were measured to confirm the formation of the supramolecular complexes. The spectra were recorded for *H*-bonded supramolecular complexes **I/An** as well as their individual components, Figure 1. The stretching vibration of the C=O was reported [62,63,64] to be at 1681 cm^−1^. The formation of the *H*-bond between the nitrogen atom of base and the COOH group of acid replaces the dimeric *H*-bonds of the 4-alkoxybenzoic acid.

Experimentally, the FT-IR spectra showed no considerable effect on the C=O vibrational strength by the *H*-bond formation of carboxylic acid. However, the stretching vibration of C=O group of the ester linkage of the azopyridine base **I** experienced a remarkable change in position as well as intensity, and the wavenumber of C=O the complex **I/A12** increased from 1740.8 to 1744.6 cm^−1^.

Moreover, it has been reported [65,66,67,68,69,70,71] that the main evidence of the H-bond formation SMC is the presence of three Fermi resonance of OH group: **A**-, **B**-, and **C**-types. The **A**-type Fermi band presented at 2917.9 to 2853.3 cm^−1^ under the C-H peaks. Moreover, the fundamental stretch vibration of the O-H (**B**-type) at 2357 cm^−1^ could be assigned to its in-plane bending vibration. On the other hand, the C-type Fermi band of the interaction between the fundamental stretching vibration of the OH and the overtone of the torsional effect appeared at 1906.2 cm^−1^.

### 3.2. Mesophase and Optical Study

**Figure 2:**
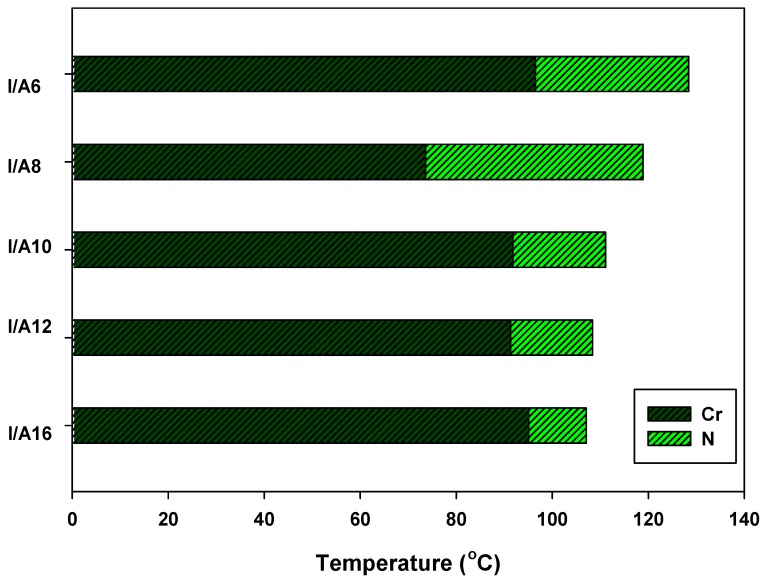
Graphical DSC transitions of supramolecular H-bonded complexes **I/An** upon second heating cycle.

**Figure 3:**
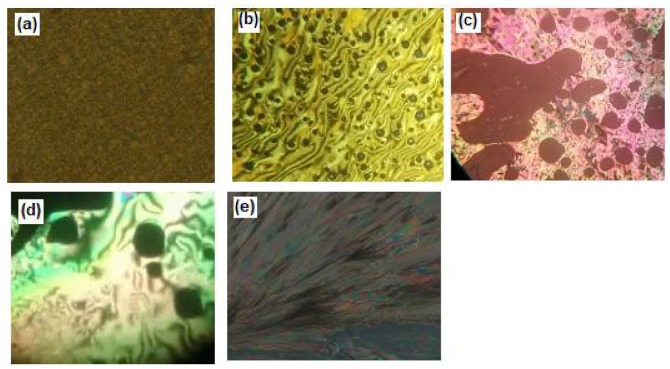
POM texture of **I/A8** (**a**) solid phase at 40.0 °C; (**b**) Schlieren N phase at 110.0 °C upon heating; (**c**) Schlieren N phase at 116.0 °C upon heating; (**d**) Schlieren N phase at 114.0 °C upon cooling; (**e**) solid phase at 20.0 °C.

**Figure 4:**
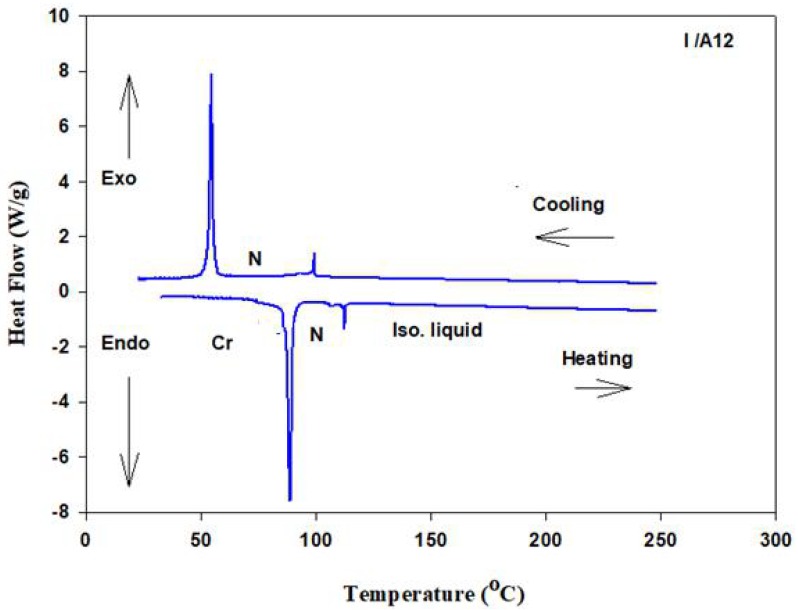
Differential scanning calorimetry (DSC) thermograms for **I/A12** upon second heating/cooling cycles.

Mesophase transition and optical behavior of the prepared supramolecular *H*-bonded complexes **I/An** were investigated. Phase transition investigations were carried out by DSC, and textures have been confirmed by POM. The temperatures and enthalpies of transitions for all binary complexes are summarized in Table 1. In addition, we studied the effect of the alkoxy chain length of the acid component on the mesophase behavior of SMHB complexes (graphically represented in Figure 2), and we will investigate the mesomorphic behaviour in terms of their geometrical and thermal parameters in the theoretical DFT part. During second heating and cooling scans, all prepared SMLHB complexes (**I/An**) showed an enantiotropic nematic phase (N) with suitable range of thermal stability. The POM investigations confirmed the Schlieren nematogenic textures (Figure 3). Representative example of DSC curves for **I/A12** was depicted in Figure 4. The azo nicotinate derivative I is non-mesomorphic (DSC thermograms attached as Supplementary Figure S1), while the 4-*n*-alkoxy benzoic acids are mesomorphic-possessing SmC and N phases depending on the length of terminal alkoxy chains [49]. Data of Table 1 and Figure 2 revealed that only enantiotropic nematic mesophase is observed for all investigated 1:1 molar supramolecular complex. Their thermal stability decreases with increment the terminal alkoxy acid chain length (n). Moreover, irregular trend was observed in melting point transitions of complexes. The 1:1 mixture of **I/A8** showed relatively higher range of nematic mesophase stability (~ 45.3 °C), and the lower range value was observed for value for **I/A16** (~ 12.0 °C). The nematic transition stability decreases with the chain length (**n**) to be in agreement with previous reports [72,73]. Thus, the terminal length and the mesogenic core of the *H*-donor component have an important role for the formation and type of the mesophase. Moreover, it had been found that that the polarity difference between *H*-donors and *H*-acceptor affects the hydrogen bonding strength and increment of the molecular anisotropy and promotes broadening of the mesophase range [32]. However, the polarity of both components of the mixture is not affected by the length of the terminal alkoxy chains.

Nematic- to isotropic transition entropies (***∆S/R***_N-I_) of present complexes **I/An** are calculated and tabulated also in Table 1. The results revealed that the entropy change (***∆S/R***_N-I_) decreased with the increments of the alkoxy chain (constant values are estimated at higher chain length (*n* = 12 and 16 carbons). The formation of less ordered N phase led to an irregular entropy- chain length relation due to the increment of the molecular end-to-end aggregations with lengthen the acid alkoxy chains.

### 3.3. DFT Theoretical Calculations

#### 3.3.1. Molecular Geometry of SMHBCs

**Figure 5:**
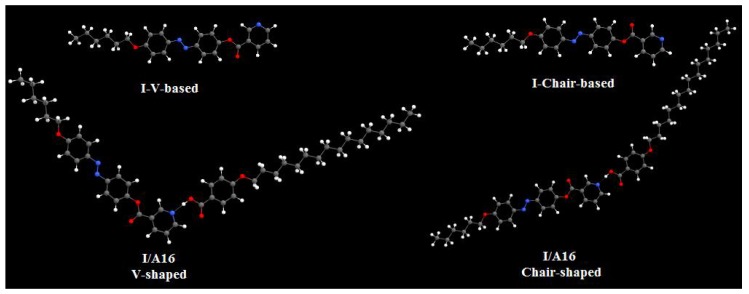
The calculated molecular geometrical conformers of the base **I**, and their SMHBCs, **I/A16.**

The prepared nicotinate base (**I**) has been proposed to exist in many conformers. We suggested two conformers (chair-based and V-based) to investigate the effect of the constitutional isomerism on the calculated thermal parameters. The theoretical estimations were carried out by DFT method at basis set B3LYP 6-31G (d,p) for all both conformers of the base (**I**) as well as the supramolecular *H*-bonded complexes **I/An**. The absence of imaginary frequencies is evidence of the geometrical stability of all H-bonded interactions. Figure 5 shows the optimum geometrical structure of the base (**I**) in both proposed conformers and their *H*-bonded complexes of *n* = 16 carbon atoms in the alkoxy chain.

Although both conformers of the base are linear and completely planar geometry, the derived SMHBCs from them are nonlinear. As shown from Figure 5, the orientation of the *N*-atom of the nicotinate moiety highly impacts the structural geometry of the *H*-bonded complex. The supramolecular complexes **I/An** exhibit nonlinear geometry with V-shaped and chair-shaped geometries.

**Scheme 4:**
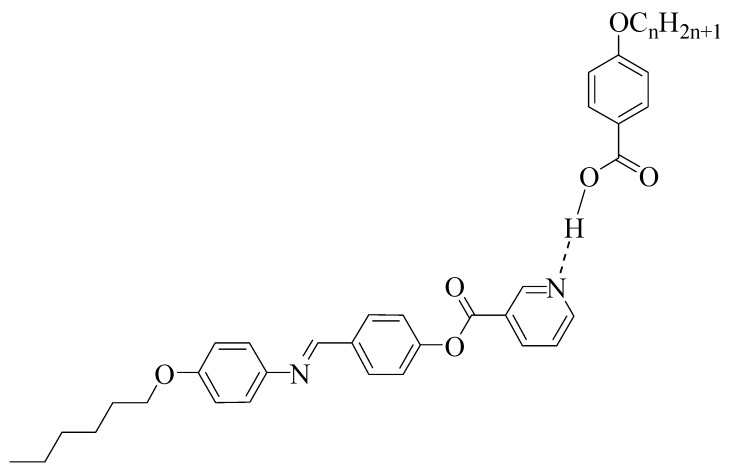
Previously prepared Schiff base.

**Figure 6:**
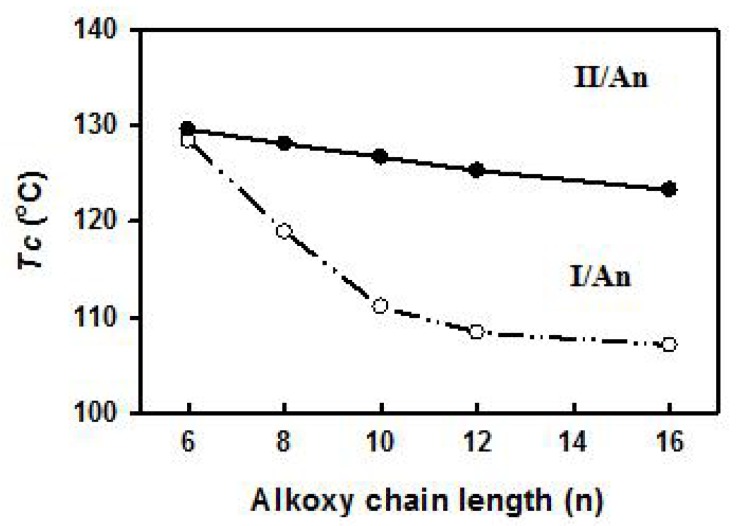
Comparison between SMHB complexes **I/An** (○) and **II/An** (●) in thermal mesophase stability.

In order to investigate the effect of changing the mesogenic core on the mesophase thermal stability (**T**_C_), a comparison was made between the present azo derivative complexes **I/An** and the our previously reported Schiff base derivative complexes, **II/An** [56]. Thermal stabilities data are illustrated in Figure 6. As can be seen from Figure 6, the replacement of Schiff base group in the base component to azo moiety resulted in decrement of the **T**c values. The analogous imino chair-shaped complexes [56], **II/An,** have been used for explaining the observed nematic mesophase, Scheme 4. The competitive intermolecular lateral and terminal molecular interactions affect the enhanced mesophase. Such interactions could be predominant due to the structural influences. Our reported analogous chair-shaped Schiff base SMHBCs enhanced the terminal interactions over the end to end ones. Consequently, these results explained formation of the less ordered nematic phase to be predominant.

#### 3.3.2. Thermal Parameters

**Figure 7:**
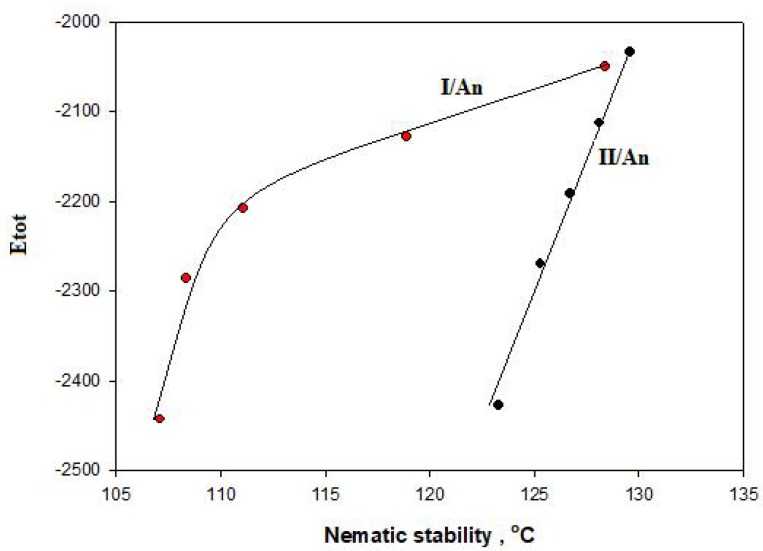
Relationship between the sum of the electronic & thermal energies of H-bonded complexes. **I/An** and **II/An** with their nematic mesophase stabilities in the chair-shaped conformers.

The estimated thermal parameters were calculated with the same method at the same set for both estimated conformers of the prepared *H*-bonded complexes (**I/An)** as well as the chair-shaped conformer of our previously reported Schiff base complexes **(II/An)**. All results are tabulated in Table 2 and reveal that the chair-shaped conformers of H-bonded complex **I/An** are more stable than the isomeric complex for the same alkoxy chain length of the acid (*n* = 6), with only 0.34513 kcal mol^−1^. This low value of the energy difference is evidence of the presence of these conformers in equilibrium. Moreover, the increment of the alkoxy chain length of the acid strongly enhances the estimated stabilities of the complexes. The longer the chain length and the more Van der Waal aggregation of the alkoxy chains, the lower the predicted energy of SMHCs. The relationship between the nematic stability of *H*-bonded complexes **I/An**, **II/An** in the chair-shaped conformers with the predicted total energy is shown in Figure 7. The figure emphasizes that the longer the alkoxy chain length has positive effect on the predicted stability, with negative effect on the nematic mesophase stability of the both *H*-bonded complexes **I/An** and **II/An**. The results showed that the there is no effect of the mesogenic core on the decrement trend. However, the decease of the nematic thermal stability could be illustrated in terms of the effect of the terminal lengths, as the chain length increases as the strength of the terminal aggregation increases and the total thermodynamic energy as well as the nematic phase stability decrease.

#### 3.3.3. Frontier Molecular Orbitals and Polarizability

**Figure 8:**
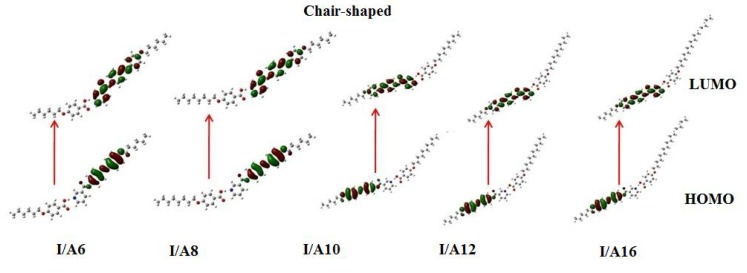
The estimated plots for frontier molecular orbitals of chair-shaped conformer of HBSMCs, **I/An.**

**Figure 9:**
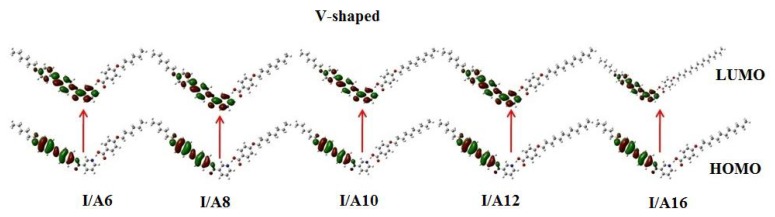
The estimated plots for frontier molecular orbitals of V-shaped conformer of HBSMCs, **I/An.**

Figure 8 and Figure 9 show the estimated plots for frontier molecular orbitals HOMO (highest occupied) and LUMO (lowest unoccupied) of both conformers of the prepared SMHDCs **I/An.** As shown from figures, it is clear that the electron densities of the sites that shared in the formation of the Frontier molecular orbitals (FMOs) were mainly localized on the nicotinate base. Moreover, there was no effect of either the orientation of the *N*-atom or the alkoxy chain length on the position of the electron densities. The energy difference between the frontier molecular orbitals could be used in the prediction of the capability of electron to transfer from HOMO to LUMO by any electron excitation process. The global softness (**S**) = **1/ΔE** is the parameter that predicts the polarizability as well as the sensitivity of the compounds for the photoelectric effects. Moreover, the higher global softness of the compounds enhanced their photoelectric sensitive as well as their polarizability. As shown from Table 3, the *H*-bonded complexes derived from the azo derivative **I** were higher in the energy gap between the FMOs than that of the Schiff base; consequently, latter was softer than that of former derived from the base **I**, for the same length of the alkoxy chain in acid moiety. Further, the lower value of the energy differences of **II/An** increased their polarizabilities.

On the other hand, the dipole moment is one of the most important factors that affect the type and behavior of the formed mesophase. From Table 3, it is obvious that the dipole moment of the V-shaped *H*-bonded complexes is higher by almost two folds than the chair-shaped. The lower dipole moment of the chair-shaped complexes is good evidence of the nematic texture of the formed mesophase. The higher dipole moment predominates side–side interaction over the end–end one; however, the lower dipole moment decreases the lateral stacking, leaving the terminal aggregation to be predominant to enhance the nematic phase. However, by comparing the dipole moment of the Schiff base mesogenic core derivative with the azo one, we could find a good illustration of the lower mesophase range of the azo compound, **I/An**, with respect to the Schiff base ones, **II/An**, Table 4. The higher dipole moment of **II/An** increases the mesophase stability and its mesophase range.

#### 3.3.4. Molecular Electrostatic Potential (MEP)

**Figure 10:**
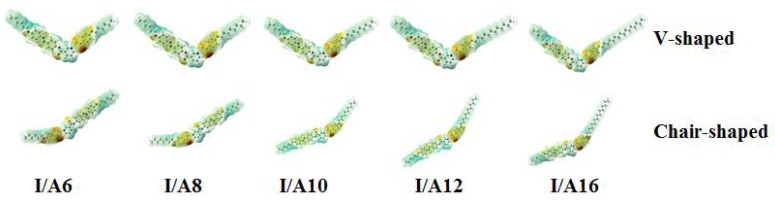
Molecular electrostatic potentials (MEP) for SMHBCs of both isomers of the nicotinate base, **I/An**.

The charge distribution map for SMHBCs of both isomers of the nicotinate base **I, I/An** and **II/An** was calculated with the same method at the same basis sets according to molecular electrostatic potential (MEP) (Figure 10). The negatively charged atomic sites (the red region) were estimated to be localized on hydrogen-bonded carboxylate moiety of the alkoxy acid, while the moiety of the base as well as the alkyl chain were predicted to show the least negatively charged atomic sites (blue regions). As shown from Figure 10, the orientation of the nitrogen atom highly impacts the orientation as well as the amount of the charges; consequently, this could be another illustration of the preference of the chair form to facilitate the terminal aggregation to enhance the nematic the mesophase. The longer alkoxy chain length of the SMHBCs **I/An** does not affect either the orientation or the amount of the charge distribution map, which could illustrate the nematic mesophase observed for all lengths **IA6-16.** On the other hand, the greater linearity of chair-shaped of the complexes **I/An** and **II/An** could illustrate the formation of the nematic mesophases of these complexes for all lengths of the alkoxy chains. This geometry permits the maximum terminal alkoxy chains aggregations to show the nematic phase rather than the V-shaped of the higher dipole moment.

## 4. Conclusions

Herein, we have synthesized and designed a new series of nonlinear geometrical-shaped supramolecular complexes liquid crystal based on Azo/ester mesogenic core. DSC and POM were carried out for mesomorphic and optical description. FT-IR spectroscopic analyses were used for chemical structure confirmation of supramolecular complex formation. The effects of the length of terminal flexible acid chains on the mesophase behavior of SMHB complexes were discussed. The results revealed that all prepared complexes showed an enantiotropic nematic phase with good mesogenic range. Moreover, the replacement of N=N by CH=N core in the base component **I** resulted in an improvement in the nematogenic range of LCs. From the DFT calculations, it could be concluded that this chair shaped isomer is more stable conformer of than the proposed V-shaped geometry. Moreover, the theoretical calculations effectively show the impact of the introduction of the azo mesogenic core instead of the Schiff base one on the mesomorphic characteristics in terms of the polarizability and the aspect ratios as well as side–side and end–end competitive intermolecular interactions.

## Figures and Tables

**Table 1:** Phase transition temperatures (°C), enthalpy of transitions (kJ/mol), and transition entropy for the supramolecular complexes **I/An.**

Complex	*T*_Cr-N_	*∆H*_Cr-N_	*T*_N-I_	*∆H*_N-I_	*∆S/R*_N-I_
**I/A6**	96.5	58.13	128.4	3.86	1.16
**I/A8**	73.6	58.37	118.9	2.55	0.78
**I/A10**	91.8	53.39	111.1	1.86	0.58
**I/A12**	91.3	53.31	108.4	1.22	0.38
**I/A16**	95.1	52.56	107.1	1.19	0.38

Abbreviations: ***T***_Cr-__N_ = crystal to nematic phase transition; ***T***_N-I_ = nematic to isotropic liquid transition. ***∆H***_Cr-N_ = crystal to nematic phase transition; ***∆H***_N-I_ = nematic to isotropic liquid transition. ***∆S/R***_N-I_ = nematic to isotropic liquid transition.

**Table 2:** Thermal parameters (Hartree/Particle) of both conformers of the H-bonded complexes I**/An.**

Conformer	Chair-Shaped					V-Shaped				
Parameter	I/A6	I/A8	I/A10	I/A12	I/A16	I/A6	I/A8	I/A10	I/A12	I/A16
**E_corr_**	0.741	0.798	0.855	0.912	1.026	0.741	0.798	0.855	0.912	1.026
**ZPVE**	−2050.616	−2129.183	−2207.750	−2286.317	−2443.451	−2050.615	−2129.182	−2207.749	−2286.316	−2443.451
**E*_tot_***	−2050.570	−2129.134	−2207.698	−2286.263	−2443.391	−2050.569	−2129.133	−2207.698	−2286.262	−2443.391
**H**	−2050.569	−2129.133	−2207.697	−2286.262	−2443.391	−2050.568	−2129.132	−2207.697	−2286.261	−2443.390
**G**	−2050.710	−2129.281	−2207.851	−2286.422	−2443.566	−2050.708	−2129.278	−2207.850	−2286.423	−2443.563

Abbreviations: ZPVE: Sum of electronic and zero-point energies; E_tot_: Sum of electronic and thermal energies; H: Sum of electronic and thermal enthalpies; G: Sum of electronic and thermal free energies.

**Table 3:** FMO Energies a.u., polarizability, α, and dipole moment μ (Debye) of geometrical conformers of HBSMCs, **I/An.**

Parameters	Chair-Shaped					V-Shaped				
	I/A6	I/A8	I/A10	I/A12	I/A16	I/A6	I/A8	I/A10	I/A12	I/A16
**E_LUMO_**	−0.098	−0.098	−0.098	−0.098	−0.098	−0.098	−0.098	−0.098	−0.098	−0.098
**E_HOMO_**	−0.225	−0.225	−0.225	−0.225	−0.225	−0.224	−0.224	−0.224	−0.224	−0.224
**ΔE_HOMO-LUMO_**	0.127	0.127	0.127	0.127	0.127	0.126	0.126	0.127	0.126	0.126
**μ Total**	3.123	3.116	3.113	3.112	3.112	5.669	5.500	5.655	5.549	5.661
**Polarizability α**	538.40	561.72	585.85	609.65	656.39	542.34	566.53	590.29	613.87	660.77

**Table 4:** FMO energies a.u., polarizability, α, and dipole moment μ (Debye) of geometrical conformers of HBSMCs, (**I,II)/A(6,8)**.

Parameters	N=N Derivatives Chair-Shaped		CH=N Derivatives Chair-Shaped	
	I/A6	I/A8	II/A6	II/A8
**E_LUMO_**	−0.098	−0.098	−0.092	−0.092
**E_HOMO_**	−0.225	−0.225	−0.213	−0.213
**ΔE_HOMO-LUMO_**	0.127	0.127	0.121	0.121
**μ Total**	3.123	3.116	4.982	4.808
**Polarizability α**	538.40	561.72	534.36	558.54

## Supplementary Materials

Supplementary Materials are available online.
