# Supplementary material for: Chair- and V-Shaped of H-bonded Supramolecular Complexes of Azophenyl Nicotinate Derivatives; Mesomorphic and DFT Molecular Geometry Aspects
Source: Molecules. 2020 Mar 26;25(7):1510. doi: 10.3390/molecules25071510 (PMC7181272; doi:10.3390/molecules25071510)
Supplement: Supplementary file 1 [file molecules-25-01510-s001.pdf]

Supplementary Data

Chair- and V- Shaped of H-bonded Supramolecular Complexes of Azophenyl Nicotinate Derivatives; Mesomorphic and DFT Molecular Geometry Aspects

Omaima A. Alhaddad[1] and Khulood A. Abu Al-Ola[1], Mohamed Hagar [2*], Hoda A. Ahmed [3*]

1. Chemistry Department, College of Sciences, Al-Madina Al-Munawarah, Taibah University, Al-Madina 30002, Saudi Arabia (OA) OHADDAD@taibahu.edu.sa; (KA) Kabualola@taibahu.edu.sa.
2. Faculty of Science, Chemistry Department, Alexandria University, Alexandria 21321, Egypt.mohamedhaggar@gmail.com (M.H.).
3. Faculty of Science, Department of Chemistry, Cairo University, Cairo 12613, Egypt, hadyelrahman@yahoo.com (H.A.A).

   * Correspondence: mohamedhaggar@gmail.com (M.H.); hadyelrahman@yahoo.com (H.A.A)

Material and methods

Nicotinic acid, *N,N′*-dicyclohexylcarbodiimide (DCC), phenol, 4-dimethylaminopyridine (DMAP) and 3-aminopyridine were purchased from Aldrich (Wisconsin, USA). All solvents used are pure grade and purchased from Aldrich (Wisconsin, USA).

TA Instruments Co. Q20 Differential Scanning Calorimeter (DSC; USA) were using for calorimetric measurements. The DSC was calibrated using the melting temperature and enthalpy of indium and lead. DSC investigation was carried out for small samples (2–3 mg) placed in aluminum pans. All measurements were achieved at a heating rate of 10°C/min in inert atmosphere of nitrogen gas (30 ml/min) and all transition recorded from the second heating scan.

Transition temperatures for the individual components and their 2:1 associated complexes, were determined by DSC, and the types of the mesophase identified by a standard polarized optical microscope (POM, Wild, Germany) attached with Mettler FP82HT hot stage. The temperature is measured by thermocouple attached to the temperature controller. Measurements were made twice, and the results have accuracy in transition temperature within ± 0.2°C.

Characterizations

Purity of **I** was checked with thin-layer chromatography using TLC and elemental analyses. The structure was confirmed by FTIR (Nicolet iS 10 Thermo scientific), and ^{1}H-NMR spectroscopy (Varian EM 350L 300 MHz spectrometer, Oxford, UK).

Computational Method and calculations

The theoretical calculations for the investigated compounds were carried out by Gaussian 09 software [1]. DFT/B3LYP methods using 6-31G (d,p) basis set was selected for the calculations. The geometries were optimized by minimizing the energies with respect to all geometrical parameters without imposing any molecular symmetry constraints. The structures of the optimized geometries had been drawn with Gauss View [2]. Moreover, the calculated frequencies were carried out using the same level of theory. The frequency calculations showed that all structures were stationary points in the geometry optimization method with none imaginary frequency.

Preparation of 4-(4-(hexyloxy)phenylazo)methyl)phenyl nicotinate (**I**).

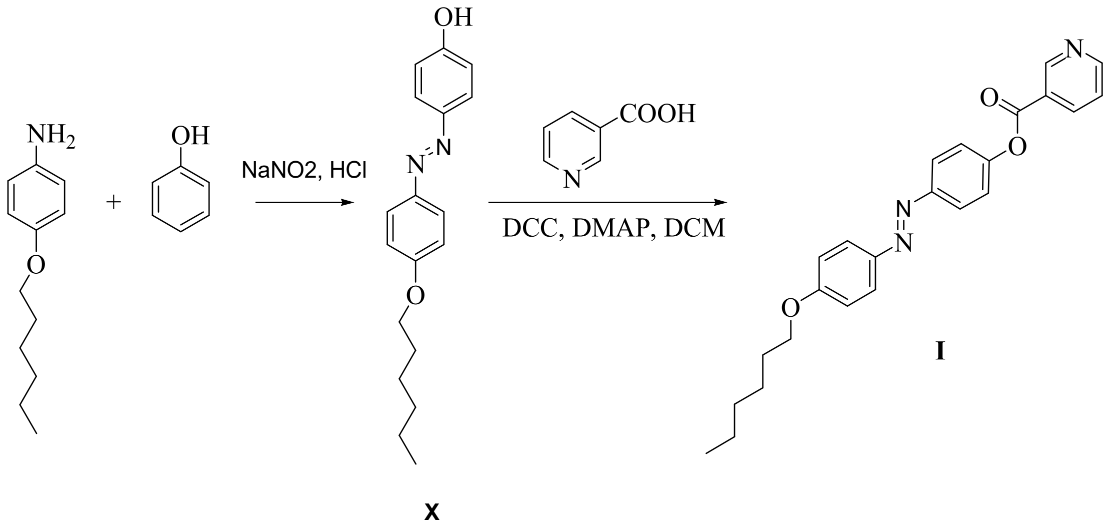

Scheme 1. Preparation of4-(4-(hexyloxy)phenylazo)methyl)phenyl nicotinate (**I**).

Synthesis of 4-((4-hexyloxyphenylazo))methyl)phenol **(x)**

This was prepared according to the method described by Zhang *et al.* [3] from 4-hexyloxyaniline and phenol. A mixture of (2.5 g, 26.5 mmol) phenol in sodium hydroxide 10% (50 ml) was cooled to 0 °C. Another cold solution of 4-hexyloxyaniline (27 mmol) in 6 molar HCl was added drop wise to a cold aqueous solution of sodium nitrite (2.0 g, 30 mmol) in H_2O (20 ml). The later solution, the diazonium solution, was added to the former, sodium phenoxide, with stirring within 10 minutes and keeping the reaction temperature below 5 °C. Subsequently, the complete precipitation of the product was performed by neutralization of the reaction mixture with sodium carbonate till pH ≈ 6. The obtained orange dye was purified by recrystallization from hot aqueous ethanol (1:1). The yield of the product was 81%.

2.3. *Synthesis of* 4-(4-(hexyloxy)phenylazo)methyl)phenyl nicotinate (**I**).

A mixture of 4–dimethylaminopyridine (DMAP) (catalytic amount) and *N*, *N′*–dicyclohexylcarbodiimide (DCC, 0.02 mole) was added to a solution of 0.01 mole 4-((4-alkoxyphenylazo))methyl)phenol (**X**) and nicotinic acid (1.23 g, 0.01 mole) in 25 ml dry methylene chloride. The reaction mixture was kept under stirring at room temperature for 72 hours. Separated byproduct, *N*,*N*-dicyclohexylurea, was filtered off. The filtrate was then evaporated till dryness. The obtained solid product was purified by recrystallization for twice from ethanol (**Scheme 1**). Thermal analysis investigations of compound I revealed to it is pure compound with sharp melting temperature.

Yield: 93 %; mp 126.2 °C, FTIR (ύ, cm^{-1}): 2919-2855 (CH_2 stretching), 1740 (C=O), 1601 (C=C), 1471 (C–O_{Asym}), 1243 (C-O $_{Sym}$).

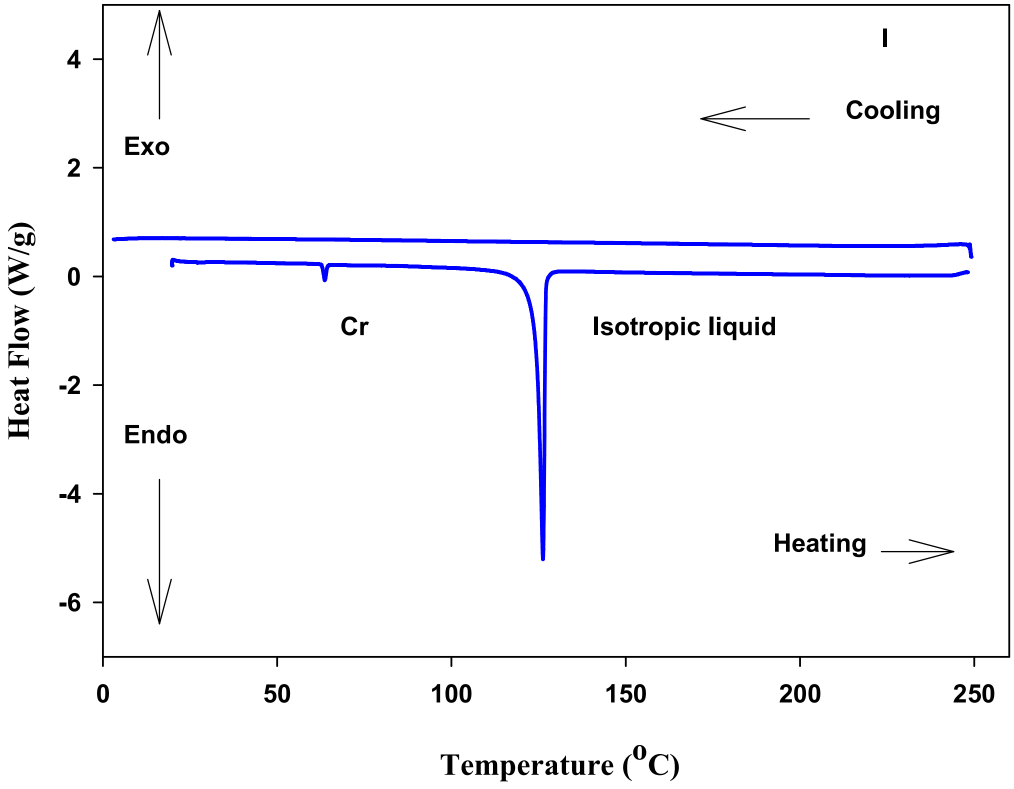

Figure S1. DSC thermograms of compound **I** upon second heating /cooling scan with rate 10 °C/min.

Table S1. Phase transition temperatures (°C), enthalpy of transitions (kJ/mol), and transition entropy for the supramolecular complexes **II/An.**

Compound	$\boldsymbol{T}_{Cr\text{-}N}$	$\boldsymbol{\Delta H}_{Cr\text{-}N}$	$\boldsymbol{T}_{N\text{-}I}$	$\boldsymbol{\Delta H}_{N\text{-}I}$	$\boldsymbol{\Delta S/R}_{N\text{-}I}$
**II/A6**	92.2	20.28	129.6	2.09	1.94
**II/A8**	91.7	52.48	128.1	3.41	3.21
**II/A10**	86.9	77.68	126.7	3.99	3.79
**II/A12**	92.5	90.23	125.3	4.04	3.88
**II/A16**	100.9	84.07	123.3	2.43	2.37

Abbreviations: $\boldsymbol{T}_{Cr\text{-}N}$= crystal to nematic phase transition; $\boldsymbol{T}_{N\text{-}I}$ = Nematic to isotropic liquid transition. $\boldsymbol{\Delta H}_{Cr\text{-}N}$= crystal to nematic phase transition; $\boldsymbol{\Delta H}_{N\text{-}I}$= Nematic to isotropic liquid transition. $\boldsymbol{\Delta S/R}_{N\text{-}I}$=Nematic to isotropic liquid transition

References

1. Frisch, M.; Trucks, G.; Schlegel, H. B.; Scuseria, G.; Robb, M.; Cheeseman, J.; Scalmani, G.; Barone, V.; Mennucci, B.; Petersson, G., Gaussian 09, revision a. 02, gaussian. *Inc., Wallingford, CT* **2009,** 200.
2. Dennington, R.; Keith, T.; Millam, J., GaussView, version 5. **2009**.
3. Song, X.; Li, J.; Zhang, S. Liq. Cryst. 2003, 30, 331–335.
